# Polycystin-2 Is Required for Chondrocyte Mechanotransduction and Traffics to the Primary Cilium in Response to Mechanical Stimulation

**DOI:** 10.3390/ijms22094313

**Published:** 2021-04-21

**Authors:** Clare L. Thompson, Megan McFie, J. Paul Chapple, Philip Beales, Martin M. Knight

**Affiliations:** 1 Centre for Predictive In Vitro Models, School of Engineering and Materials Science, Queen Mary University of London, London E1 4NS, UK; m.r.mcfie@qmul.ac.uk (M.M.); m.m.knight@qmul.ac.uk (M.M.K.); 2 Centre for Endocrinology, William Harvey Research Institute, School of Medicine and Dentistry, Queen Mary University of London, London EC1M 6BQ, UK; j.p.chapple@qmul.ac.uk; 3 Genetics and Genomic Medicine, UCL Great Ormond Street Institute of Child Health, London WC1N 1EH, UK; p.beales@ucl.ac.uk

**Keywords:** Polycystin, Polycystin-1, Polycystin-2, cilia, strain, cartilage, chondrocyte, mechanotransduction

## Abstract

Primary cilia and associated intraflagellar transport are essential for skeletal development, joint homeostasis, and the response to mechanical stimuli, although the mechanisms remain unclear. Polycystin-2 (PC2) is a member of the transient receptor potential polycystic (TRPP) family of cation channels, and together with Polycystin-1 (PC1), it has been implicated in cilia-mediated mechanotransduction in epithelial cells. The current study investigates the effect of mechanical stimulation on the localization of ciliary polycystins in chondrocytes and tests the hypothesis that they are required in chondrocyte mechanosignaling. Isolated chondrocytes were subjected to mechanical stimulation in the form of uniaxial cyclic tensile strain (CTS) in order to examine the effects on PC2 ciliary localization and matrix gene expression. In the absence of strain, PC2 localizes to the chondrocyte ciliary membrane and neither PC1 nor PC2 are required for ciliogenesis. Cartilage matrix gene expression (*Acan*, *Col2a*) is increased in response to 10% CTS. This response is inhibited by siRNA-mediated loss of PC1 or PC2 expression. PC2 ciliary localization requires PC1 and is increased in response to CTS. Increased PC2 cilia trafficking is dependent on the activation of transient receptor potential cation channel subfamily V member 4 (TRPV4) activation. Together, these findings demonstrate for the first time that polycystins are required for chondrocyte mechanotransduction and highlight the mechanosensitive cilia trafficking of PC2 as an important component of cilia-mediated mechanotransduction.

## 1. Introduction

The primary cilium is a microtubule-based signaling organelle essential for numerous cell signaling pathways and biological processes that include differentiation, proliferation, inflammation, and mechanotransduction (for a review, see [1]). A single immotile cilium is expressed by the majority of cells in the body, including chondrocytes, which are the cells within articular cartilage. This connective tissue covers the bone surfaces of the joint and functions to distribute load, thereby protecting the underlying bone from high stresses. Chondrocytes are responsible for maintaining the health of the tissue and regulate cartilage extracellular matrix turnover in response to mechanical stimuli [2,3,4]. Disruption of this process results in cartilage degeneration, as seen in cartilage disease such as osteoarthritis (OA, for a review, see [5]).

The primary cilium plays an important role in cartilage health and disease. During development, the mutation of cilia-related genes such as IFT88 and Kif3a affect embryonic patterning largely due to disruptions in hedgehog signaling [6,7]. Cartilage-specific deletion of IFT88 affects long bone formation and matrix remodeling within the articular cartilage, such that a mechanically deficient tissue is formed [8,9]. Cartilage thinning and other abnormalities are observed in Bardet–Biedl Syndrome mutant mice, which is consistent with early signs of OA [10]. More recently, Coveney et al. reported that tissue-specific deletion of IFT88 in adolescence results in cartilage thinning associated with an increase in spontaneous arthritis later in adulthood [11]. Moreover, these mice exhibit a greater level of joint damage following the surgical induction of OA, which is indicative of a chondroprotective role for the cilium through modulation of mechanotransduction [11].

The primary cilium has long been recognized as a mechanosignaling organelle that is important in the process of mechanotransduction. In kidney epithelium, the cilium projects from the apical cell surface such that bending of the ciliary axoneme in response to fluid flow initiates a calcium signaling cascade that regulates cellular function [12,13,14,15]. This response has been attributed to the ciliary functions of the polycystin family proteins. Polycystin-2 (PC2) is an integral, multi-pass membrane protein, and it is a member of the transient receptor potential polycystic (TRPP) family, which functions as a non-selective cation channel [16]. PC2 interacts with polycystin-1 (PC1) to form a complex whose cilia localization is implicated in mechanosensitive calcium signaling and the maintenance of normal renal tubular development [17,18]. Mutations in *pkd1* or *pkd2*, which encode PC1 and PC2 respectively, result in autosomal dominant polycystic kidney disease (ADPKD), which is one of the most common cilia-related pathologies [19]. While recent work from Clapham and colleagues [20] questions the precise sequence of ciliary Ca^2+^ signaling events, subsequent studies confirm that PC2 ciliary localization is required to prevent cyst formation in polycystic kidney disease (PKD) mouse models [18].

Chondrocyte primary cilia are predominantly found within a deep ciliary pocket, are short (1–2 µm), and do not commonly project out from the cell surface [21,22]. Yet, in vitro studies have established a role for the cilium in chondrocyte mechanosignaling. In chondrocytes isolated from the Oak Ridge Polycystic Kidney disease (ORPK) mouse, cilia loss prevents the upregulation of cartilage matrix production in response to mechanical stimulation [23]. Wann et al. observed a failure to generate an appropriate mechanosensitive calcium signal in these cells, which is necessary for the upregulation of proteoglycan synthesis and attributes this to altered processing of the PC1 C-terminal tail region [24]. However, cilia loss did not disrupt mechanosensitive ATP release, which occurs upstream of these events, suggesting that cilium is required for signal transduction but not necessarily mechanosensation in these cells [25]. In other studies, knockdown of KIF3A in a chondrocytic cell line similarly altered the transcriptional response to mechanical stimulation, including the regulation of genes encoding aggrecan and collagen type II [26]. Furthermore, He et al. reported a role for the cilium in the mechanosensitive regulation of genes encoding catabolic enzymes [27].

In this study, we further investigate the role of polycystins in chondrocyte cilia maintenance and function. PC1 and PC2 interaction occurs through the cytoplasmic C-terminal region of these proteins and is reportedly important not only for channel function but also cilia localization [28,29,30]. We hypothesize that PC2 and its ciliary localization is an important component of the anabolic signaling pathway, regulating downstream changes in matrix gene expression in response to mechanical stimuli.

## 2. Results

### 2.1. PC1 and PC2 Are Required for Anabolic Gene Expression in Response to Cyclic Tensile Strain

**Figure 1:**
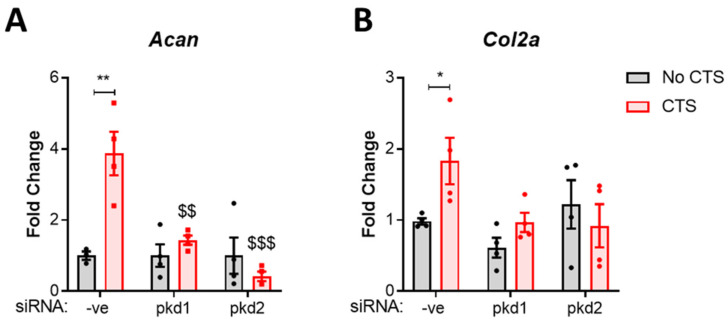
Polycystin-1 and polycystin-2 are required for the anabolic response to strain. Immortalized murine chondrocytes were treated with siRNA for *pkd1* and *pkd2* to deplete Polycystin-1 and Polycystin-2 expression and a negative control siRNA (-ve). Cells were subjected to 10% cyclic tensile strain (CTS) for 1 h at 0.33 Hz. Changes in gene expression for (**A**) aggrecan (*acan*) and (**B**) collagen type II (*col2a*) were quantified by qRT-PCR. Samples were normalized to GAPDH and expressed as a fold change relative to the -ve control (Statistics: two-way ANOVA with Tukey’s multiple comparisons test, *n* = 4). Significance is displayed relative to the no CTS -ve control as * = *p* < 0.05 and ** = *p* < 0.01, whereas $ denotes significance relative to the CTS -ve siRNA ctrl group where $$ = *p* < 0.01 and $$$ = *p <* 0.001.

Firstly, the role of PC1 and PC2 in the anabolic response to strain was assessed. The expression of *pkd1* and *pkd2*, which encode PC1 and PC2, respectively was depleted by siRNA-mediated knockdown in the same immortalized wild-type murine fibroblast-like chondrocyte cell line used by Wann et al. [23,31]. Then, chondrocytes were subjected to mechanical stimulation in the form of 10% cyclic tensile strain (CTS) for 1 h at 0.33 Hz and the effects on matrix gene expression examined. In the control group (-ve siRNA), CTS resulted in a significant increase in aggrecan (Acan, *p* = 0.0014, Figure 1A) and collagen type II (Col2a, *p* = 0.0421, Figure 1B) gene expression by 4-fold and 2-fold, respectively. No differences were observed in Acan or Col2a expression in the absence of strain. However, both *pkd1* and *pkd2* siRNA completely inhibited the mechanosensitive upregulation of these genes, confirming the importance of their expression in this response.

### 2.2. PC2 Localizes to the Chondrocyte Primary Cilium

**Figure 2:**
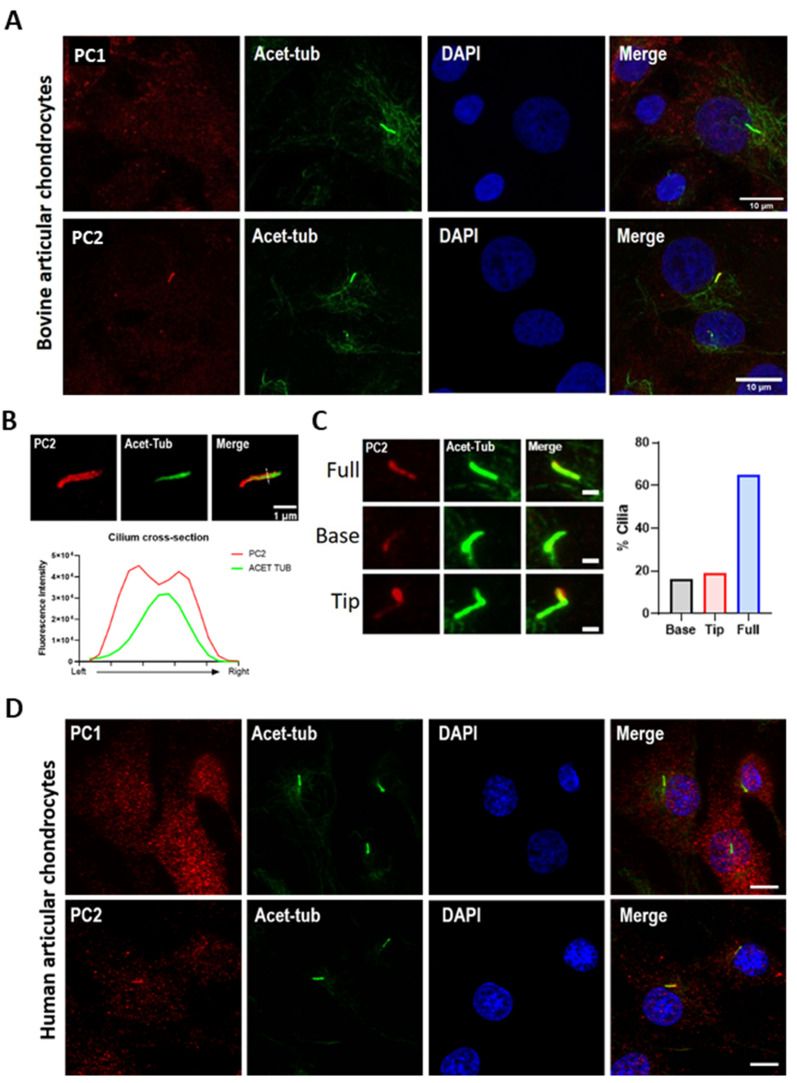
Polycystin-2 localizes to the chondrocyte primary cilium. (**A**) Primary bovine articular chondrocytes were immunolabeled for Polycystin-1 (PC1, red) or Polycystin-2 (PC2, red), primary cilia were labeled for acetylated tubulin (acet-tub, green), and nuclei were counterstained with DAPI (blue). Scale bar = 10 µm. (**B**) Representative structured illumination microscopy (SIM) image of PC2 labeled cilium. PC2 (red) and acet-tub (green) accompanied by a fluorescence intensity plot of a cross-section through the axoneme. Scale bar = 1 µm. (**C**) Representative image of cPC2 cilia distribution accompanied by quantifcation (*n* = 100 cilia). Scale bar= 1 µm. (**D**) Primary human articular chondrocytes were immunolabeled for PC1 (red) or PC2 (red), primary cilia were labeled for acet-tub (green), and nuclei were counterstained with DAPI (blue). Scale bar = 10 µm.

We next examined PC1 and PC2 protein localization using primary bovine articular chondrocytes isolated from healthy tissues (Figure 2A). PC2 localization was found to be enriched within the cilium co-localizing with the ciliary marker acetylated α-tubulin (Figure 2A). Donor variability in the proportion of ciliated cells exhibiting PC2 localization was observed from 30 to 70% (Figure S1). Overall, PC2 cilia localization was observed in 50% of cilia. Structured illumination microscopy (SIM) imaging of chondrocyte cilia revealed that staining was localized to the ciliary membrane (Figure 2B). PC2 localized along the full length of the axoneme with varying intensity. No preferential distribution toward either the base or tip was observed (Figure 2C). Consistent with previous reports [23], ciliary localization of PC1 was not observed (Figure 2A).

PC2 ciliary localization was also observed in primary human articular chondrocytes (Figure 2D) and, as previously reported in immortalized wild-type murine fibroblast-like chondrocytes [23]. Similarly, PC1 cilia localization was not observed in human cells (Figure 2D). In murine chondrocytes, up to 50% of cells typically exhibited PC2 ciliary staining similar to that in bovine and human cells (Figure S2). Interestingly, while cilia prevalence was significantly reduced in chondrocytes isolated from the ORPK mouse (IFT88^ORPK^), amongst the small number of cilia that were able to form, a similar proportion of PC2 localization was observed (Figure S2).

### 2.3. PC2 Ciliary Localization Is Dependent on PC1

**Figure 3:**
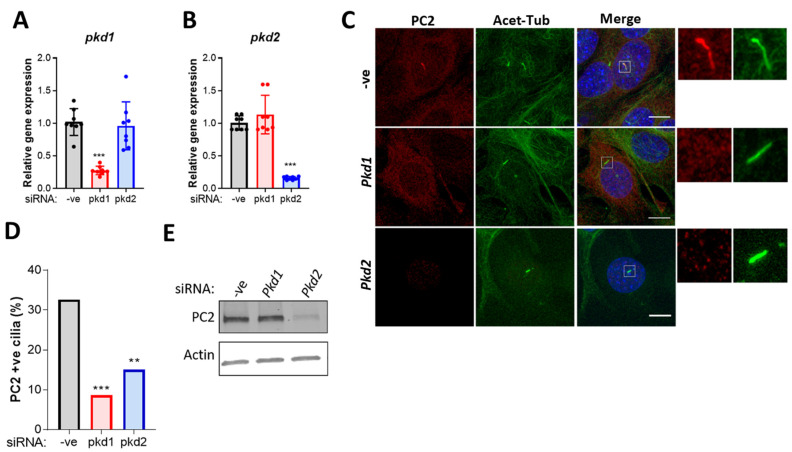
Immortalized murine chondrocytes were treated with siRNA for *pkd1* and *pkd2* and a negative control siRNA (-ve). The effects on (**A**) *pkd1* and (**B**) *pkd2* gene expression were determined. Samples were normalized to GAPDH and expressed as a fold change relative to the -ve control. Data represents mean ± SD (Statistics: One-way ANOVA with Tukey’s multiple comparisons test, *n* = 8). (**C**) Cells were labeled for polycystin-2 (PC2, red) and acetylated α-tubulin (acet-tub, green) and nuclei were counter-stained for DAPI (blue). Scale bar 10 µm. (**D**) The proportion of PC2-positive cilia was quantified (statistics: fishers exact test, *n* > 100 cilia). (**E**) Representative Western blot to determine changes in total PC2 expression (*n* = 3). Significance is displayed as: ** = *p* < 0.01, *** = *p* < 0.001.

Several studies suggest that PC1 and PC2 traffic to the cilium together and enter the ciliary compartment as a complex [32,33,34,35]. However, PC2 contains a discrete ‘RVxP’ ciliary targeting signal, and ciliary localization can be observed in cells that have undergone PC1 inactivation, indicating that complex formation is not essential [36,37]. Therefore, we examined the effects of *pkd1* siRNA on PC2 ciliary localization in murine chondrocytes (Figure 3A–C). We report that *pkd1* siRNA results in a significant decrease in the proportion of PC2 positive cilia (Figure 3D,E, *p* < 0.0001). Loss of ciliary PC2 localization was not accompanied by a change in PC2 protein expression nor accumulation of this protein at the ciliary base (Figure 3C,E). These data suggest that PC1 is required for PC2 trafficking and ciliary localization and supports the hypothesis that these proteins traffic to the cilium together in murine chondrocytes.

### 2.4. PC2 Ciliary Localization Is Increased in Response to Mechanical Stimulation and Requires PC1

**Figure 4:**
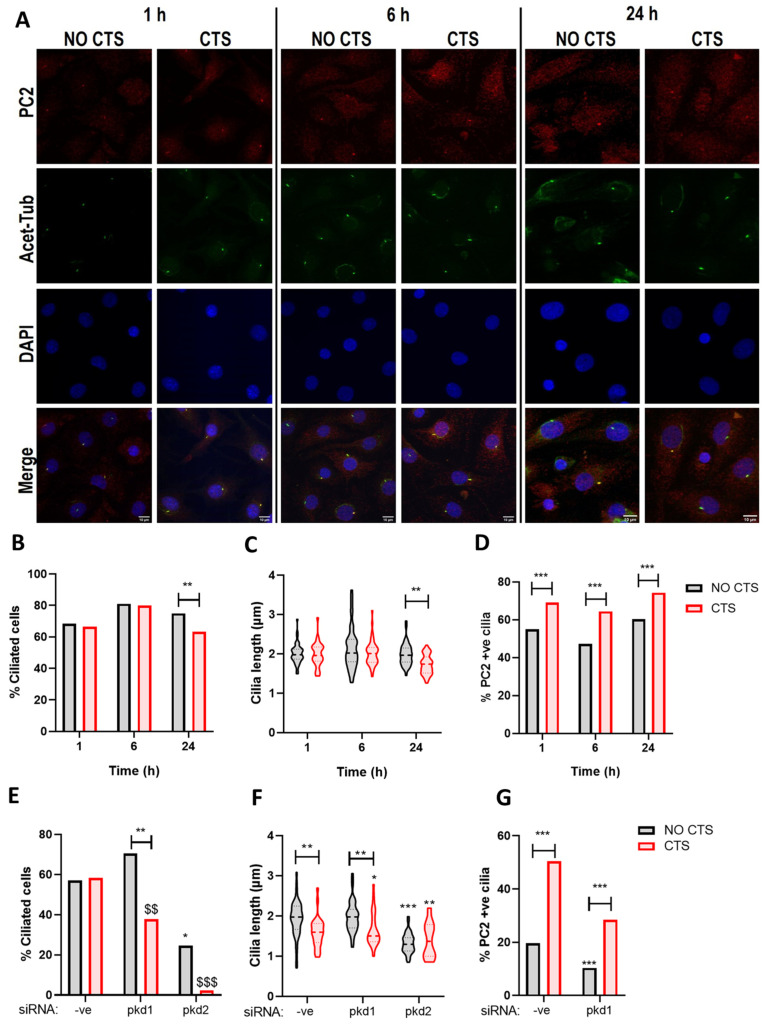
Polycystin-2 ciliary localization is increased in response to strain. Bovine articular chondrocytes were cultured on elastomeric membranes and subjected to 10% cyclic tensile strain (CTS) for 1, 6, and 24 h at 0.33 Hz. (**A**) Chondrocytes were immunolabeled for polycystin-2 (PC2, red), primary cilia were labeled for acetylated α-tubulin (acet-tub, green) and nuclei were counterstained with DAPI (blue). Scale bar = 10 µm. (**B**) Cilia length (*n* = 48 fields, two-way ANOVA), (**C**) cilia prevalence (*n* > 800 cells, chi-squared test), and (**D**) the proportion of PC2-positive cilia (*n* > 600 cells, chi-squared test) were quantified. Immortalized murine chondrocytes were transfected with -ve control and *pkd1* siRNA was then subjected to 10% CTS for 1 h. (**E**) The proportion of ciliated cells (*n* > 500 cells, chi squared test) and (**F**) cilia length were quantified (*n* > 48 fields, two-way ANOVA). (**G**) Chondrocytes were immunolabeled for PC2 (red) and acet-tub (green), and the proportion of PC2 positive cilia was quantified (*n* > 200 cilia, chi squared test). Significance is displayed relative to the unstrained control as: * = *p <* 0.05, ** = *p* < 0.01 and *** = *p* < 0.001 whereas $ denotes significance relative to the CTS -ve siRNA ctrl group where $$ = *p* < 0.01 and $$$ = *p <* 0.001.

Mechanical stimuli influence ciliary structure and promote cilia disassembly in multiple cell types including chondrocytes [38,39,40]. The mechanisms governing the regulation of cilia structure are intrinsically linked to protein traffic through the ciliary compartment. Therefore, we examined the effects of uniaxial cyclic tensile strain (CTS) on both ciliation and PC2 localization. Bovine articular chondrocytes were cultured on elastomeric membranes and subjected to 10% CTS for 1, 6, and 24 h (Figure 4A). While some variability in ciliation was observed over the culture period, the proportion of ciliated cells was not altered by CTS at 1 h and 6 h (Figure 4B), nor was there any significant effect on cilia length (Figure 4C). However, both cilia prevalence and length were significantly reduced following CTS for 24 h (Figure 4B,C, *p* = 0.0079 and *p* = 0.0092 respectively). A significant increase in the proportion of PC2 positive cilia was observed from 58.7% in the No CTS control group to 70.1% in cells subjected to CTS at 1 h (*p* = 0.0078); this increased ciliary localization was maintained, but it did not increase further with increasing load duration (Figure 4D).

To explore the role of PC1-dependent ciliary trafficking of PC2 in this response, *pkd1* gene expression was depleted in immortalized murine chondrocytes, and the cells were subjected to 10% CTS for 1 h. In the absence of strain, *pkd1* siRNA significantly increased cilia prevalence (Figure 4E, *p* < 0.0001) but reduced the overall proportion of PC2 positive cilia (Figure 4G, *p* < 0.0001). There was no effect of pkd1 siRNA on cilia length in the absence of strain (Figure 4F, *p* = 0.9876). While CTS did not influence ciliation in the control siRNA group (-ve), a significant reduction in ciliation was observed in response to CTS for cells treated with *pkd1* siRNA (Figure 4E, *p* < 0.0001). A significant reduction in cilia length was observed in both -ve and *pkd1* siRNA treated cells following CTS (Figure 4F). While CTS significantly increased PC2 ciliary localization in the -ve siRNA control group (*p* < 0.0001), this response was attenuated by *pkd1* siRNA (Figure 4G). These data suggest that PC1, at least partially, mediates PC2 ciliary localization in the presence of strain. Of note, *pkd2* siRNA resulted in a significant decrease in cilia length and prevalence in unstrained cells, which was further exacerbated upon the application of CTS such that less than 10% of cells exhibited a cilium (Figure 4E,F).

### 2.5. The Mechanosensitive Increase in PC2 Cilia Localization Is Not Dependent upon ATP Release

**Figure 5:**
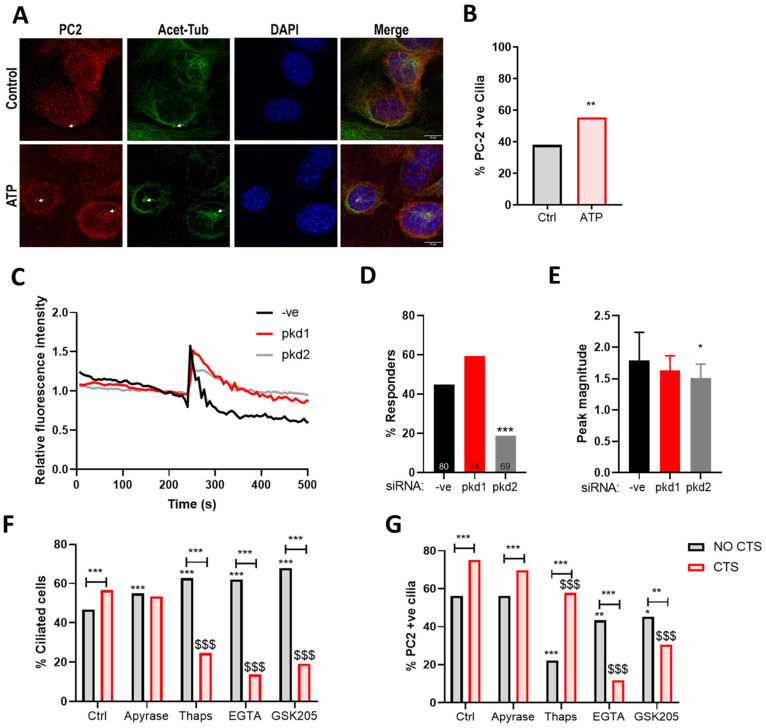
Polycystin-2 strain-dependent cilia localization is dependent on TRPV4-dependent Ca^2+^ signaling. (**A**) Immortalized murine chondrocytes were treated with 100 µM ATP and labeled for polycystin-2 (PC2, red) acetylated tubulin (green) and DAPI (blue). Scale bar = 10 µm. (**B**) The proportion of PC2-positive cilia was quantified (*n* > 100 cilia, chi-squared test). (**C**) Representative Ca^2+^ transients in murine chondrocytes treated with ATP. (**D**) The proportion of chondrocytes exhibiting a Ca^2+^ transient following transfection with *pkd1*, *pkd2* or -ve control siRNA (*n* = 100 cells, Chi squared test). (**E**) Peak magnitude of identified Ca^2+^ transients (*n* > 13 transients, one-way ANOVA with post hoc Tukey’s multiple comparisons. Immortalized murine chondrocytes were pre-treated with Apyrase, Thapsigargin (Thaps), ethylene glycol-bis(β-aminoethyl ether)-N,N,N′,N′-tetraacetic acid (EGTA), and GSK205 then subjected to 10% cyclic tensile strain (CTS) for 1 h at 0.33 Hz. The proportion of (**F**) ciliated cells (*n* > 700 cells, chi-squared test) and (**G**) PC2 positive cilia were quantified (*n* > 350 cilia, chi-squared test). Significance is expressed relative to the No CTS Ctrl, unless otherwise indicated * = *p* < 0.05, ** = *p* < 0.01, *** = *p* < 0.001. $ denotes significance relative to the CTS ctrl group where $$$ = *p* < 0.001.

Upon mechanical stimulation, chondrocytes activate a purinergic Ca^2+^ signaling response, which requires a functional cilium and regulates matrix gene expression [23,25,41]. PC2 ciliary trafficking has been linked to the modulation of intracellular Ca^2+^ levels [36,42]. Therefore, the role of ATP release was examined in the mechanosensitive PC2 cilia trafficking response. Consistent with previous reports [36], ATP treatment (100 µM) triggers a robust Ca^2+^ signaling response in chondrocytes (Figure 5A–C) accompanied by a significant increase in the proportion of PC2 positive cilia (Figure 4A,B). Purinergic Ca^2+^ signaling was found to be attenuated by *pkd2* (*p* < 0.0001) but not *pkd1* siRNA.

To examine the role of mechanosensitive ATP release, chondrocytes were pre-treated for 3 h with the ATP-diphosphohydrolase, apyrase (10 U/mL) and then subjected to 10% CTS for 1 h. CTS resulted in a significant increase in the proportion of ciliated cells (*p* < 0.0001); this response was completely inhibited by apyrase (Figure 5A). However, a significant increase in the proportion of cells exhibiting PC2 cilia localization was still observed following CTS, indicating that ATP release is not necessary for this response (Figure 5B).

### 2.6. The Mechanosensitive Increase in PC2 Cilia Localization Is Dependent upon TRPV4 Activation and the Influx of Extracellular Ca^2+^

To explore the role of mechanosensitive Ca^2+^ signaling in this response, we inhibited both intracellular and extracellular calcium release in the presence of CTS using thapsigargin (1 µM) and the Ca^2+^ chelator ethylene glycol-bis(β-aminoethyl ether)-N,N,N′,N′-tetraacetic acid (EGTA, 10 µM), respectively. Cells were pre-treated for 3 h and then subjected to 10% CTS for 1 h. Both thapsigargin and EGTA influenced ciliation at baseline, significantly increasing the proportion of ciliated cells (*p* < 0.0001, Figure 5F). Interestingly, both compounds significantly decreased the proportion of ciliated cells in response to CTS (*p* < 0.0001, Figure 5F). However, while both compounds resulted in a reduction in PC2 cilia localization in the No CTS control group (*p* < 0.0001, Figure 5G), only EGTA was found to inhibit the mechanosensitive increase in PC2 cilia localization in response to strain (Figure 5G), indicating that this phenomenon is dependent on the influx of extracellular Ca^2+^.

Previously, we show that CTS activates transient receptor potential cation channel subfamily V member 4 (TRPV4), which in turn regulates the cytoplasmic deacetylase histone deacetylate 6 (HDAC6) to direct mechanosensitive changes in chondrocyte cilia structure [42]. Therefore, we inhibited TRPV4 function (GSK205, 10 µM) and examined the effects on ciliation and PC2 cilia localization. TRPV4 inhibition mimicked the response observed with EGTA such that the mechanosensitive increase in PC2 cilia localization was inhibited (Figure 5F,G).

## 3. Discussion

In the current study, we demonstrate for the first time that the TRPP channel protein PC2 is required for the anabolic response to mechanical strain in chondrocytes. In the absence of PC1 or PC2, the mechanosensitive gene expression of aggrecan and collagen type II is inhibited, supporting a role for the ciliary trafficking of PC2 in chondrocyte mechanotransduction. PC2 localizes to the chondrocyte ciliary membrane, and this localization is increased upon the application of strain. Mechanosensitive PC2 ciliary trafficking requires PC1 and occurs downstream of TRPV4 activation and influx of extracellular Ca^2+^ ions.

In situ chondrocytes exhibit a rounded morphology and are embedded within a dense cartilage matrix comprised of highly hydrated proteoglycans bounded by an organized collagen network. Consequently, during physiological joint loading, chondrocytes experience a variety of mechanical or physicochemical stimuli such as compressive, tensile, and shear strain in addition to fluid flow, electrical streaming potentials, and changes in pH and osmolarity (for a review, see [43]). It is well known that the nature of the mechanical loading, including the magnitude and frequency, influences aspects of cell behavior, and hence, it is possible that these factors may also modulate mechanosensitive ciliary polycystin trafficking as reported here.

Cellular strain within the articular cartilage varies with the depth of the tissue (0–20% compressive strain) such that the highest strains are experienced at the articular surface [44]. In the current study, we examined the effects of tensile strain on chondrocytes in 2D culture. This model is advantageous, as it provides a sensitive and highly reproducible system in which to reliably measure changes in cilia trafficking and length. The current loading regime was chosen based upon previous studies that demonstrate that 10% tensile strain at 0.33 Hz results in membrane depolarization and the regulation of chondrocyte gene expression [45]. While it may not reflect the fully complexity of physiological loading in situ, the effects of tensile strain on chondrocyte function have been widely studied in 2D culture [27,45,46,47] and reproduce phenomena observed in response to more physiological 3D compression in terms of regulation of matrix gene expression, cilia expression, and inflammatory signaling [3,38,45,48].

Wann et al. have demonstrated that the chondrocyte cilium is required for signaling downstream of mechanosensitive ATP release and suggest that defective PC1 processing leads to disrupted signaling in cilia mutant cells [23]. The current study demonstrates the importance of both PC1 and PC2 function in the chondrocyte mechanotransduction response. Increased ciliary localization of PC2 was observed in the absence of strain following stimulation with exogenous ATP, which is consistent with previous studies in kidney epithelium that indicate PC2 ciliary trafficking can be regulated by the modulation of intracellular Ca^2+^ levels [36]. Consistent with this, treatment with thapsigargin, a non-competitive inhibitor of the sarco/endoplasmic reticulum Ca^2+^ ATPase, significantly reduced PC2 ciliary localization in the absence of strain (Figure 5B). By contrast, the mechanosensitive increase in PC2 ciliary localization was found to be strongly dependent on the influx of extracellular Ca^2+^ ions via stimulation of TRPV4. TRPV4 is a non-selective cation channel activated by changes in temperature, hyperosmotic conditions, and mechanical stress (for a review, see [49]). Intriguingly, these data suggest the mechanisms responsible for regulating ciliary trafficking in the absence or presence of mechanical stimulation are distinct, with the response to strain being strongly dependent upon the activity of stretch-activated ion channels present in the plasma membrane such as TRPV4. These findings are supported by our previous studies that suggest that the pharmaceutical modulation of cilia structure has a differential effect on chondrocyte inflammatory signaling in strained and unstrained cells [50].

A role for TRPV4 in chondrocyte mechanotransduction is well established. TRPV4 inhibition with GSK205 inhibits chondrocyte matrix production in response to compressive mechanical stimulation, while the agonist GSK1016790A (GSK101) promotes matrix production in the absence of stimulation [51]. Moreover, TRPV4-deficient mice have an increased risk of obesity-related OA [52]. TRPV4 activation can trigger ATP release in multiple cell types, including chondrocytes [53,54,55]. In porcine chondrocytes, GSK101 mimics the effects of hypotonic media and stimulates ATP efflux [56]. Therefore, we suggest that mechanosensitive TRPV4 activation likely occurs upstream of ATP release, but it modulates PC2 ciliary localization via alterations in Ca^2+^ signaling.

Understanding the complex role of the primary cilium in articular cartilage in vivo is confounded by the effects of cilia loss on skeletal development. Most recently, Coveney et al. used the cartilage-specific inducible deletion of IFT88 to remove cilia in mouse joints after skeletal maturity [11]. These studies confirmed a role for the cilium in the mechanosensitive regulation of matrix production, with defects in cartilage formation/maintenance only apparent in regions of the joint subject to higher levels of loading in aged mice [11]. In the current study, loss of *pkd1*/*pkd2* expression did not result in a change in the regulation of matrix gene expression in the absence of strain, but it rather inhibited mechanosensitive matrix gene expression (Figure 1). *Pkd1* is expressed in both developing and mature cartilage, and in mice with targeted disruption of *pkd1* expression, cranial, facial, axial, and long bone formation defects have been observed [57]. There is significant evidence for a role for PC1 in mechanotransduction in bone, where it promotes the mechanosensitive expression of osteoblastic differentiation markers through interactions with Yes-associated protein/WW domain containing transcription regulator 1 (YAP/TAZ) [58,59], the calcineurin/nuclear factor of activated T cells (NFAT) pathway [60], β-catenin [61], and Janus kinase2/signal transducer and activator of transcription 3 (JAK/STAT3) signaling [62]. However, a role for PC2 in skeletal physiology is less clear. No obvious skeletal patterning defects have been reported for *pkd2*^−/−^ mice. However, these mutations are embryonic lethal due to severe cardiac defects and renal failure, so skeletal patterning defects cannot be ruled out [63]. Indeed, in zebrafish, polycystin proteins are reported to play a direct role in in the modulation of collagen expression or assembly [18]. Meanwhile, pkd2 inactivation specifically in mature mouse osteoblasts leads to osteopenia and these mice exhibit reduced expression of several osteoblast-specific genes markers [37]. Therefore, the in vivo ciliary function of PC2 may similarly prove to be more subtle and contribute to the chondroprotective function of this organelle observed in response to joint trauma [11].

Due the ubiquitous expression of PC2 within the plasma membrane, endoplasmic reticulum, and cilium, it is difficult to distinguish the importance of the ciliary localization of this protein. Walker et al. reported that in mice carrying a non-ciliary localizing, yet channel-functional, PC2 mutation, embryonic renal cysts are still observed such that these mice are indistinguishable from mice completely lacking PC2 [48]. While PC2 has the capacity to regulate Ca^2+^ signaling in response to ATP, we report that PC1 is not required for this function, which suggests PC1-dependent PC2 cilia trafficking is likewise not required for this response, either.

Ciliary localization of PC1 was not observed in chondrocytes of human, mouse, or bovine origin. PC2 accumulation at the ciliary base was not observed in cells treated with *pkd1* siRNA, suggesting that PC1 interaction mediates the initial transport to the cilium in response to strain but not necessarily cilia entry of PC2. PC1 and PC2 interact at a ratio of 1:3 [64]; therefore, it may be that endogenous PC1 cilia localization is below the threshold of detection in our model. Alternatively, PC1 is reported to sense cytosolic Ca^2+^ levels through binding to calmodulin (CaM), which could be important for the cilia trafficking of PC1/PC2 in response to strain [42]. CaM binding reportedly inhibits PC1/PC2 channel activity [40]; however, Lui et al. showed that in renal epithelium, PC2 but not PC1 is a required subunit for the ion channel in the primary cilium [65]. Indeed, TRPV4 and PC2 can reportedly form a polymodal sensory channel complex within the cilium that is required for cilia mediated calcium transients [66]. In mesenchymal stem cells, TRPV4 modulates the mechanotransduction response to fluid shear in part via the primary cilium, and the concentration of TRPV4 is observed at the ciliary base [62]. TRPV4 localization was not investigated in the current study; however, ciliary localization has been reported in chondrocytes [50,67]. Therefore, increased trafficking of PC2 under load could function to promote further interaction between these proteins within the cilium itself as part of a positive feedback loop. This highlights an intriguing prospect that targeting ciliary trafficking to promote PC2 localization could in essence generate a “mechanically primed” cilium to enhance the downstream mechanotransduction events in response to subsequent stimuli.

Intriguingly, robust cilia disassembly was observed in *pkd2* siRNA treated cells in response to strain, suggesting that rather than a direct signaling role in matrix gene expression, this protein may function to protect cilia from mechanically induced disassembly. In osteoclasts, ciliary length regulation is tightly coupled to the activity of adenylyl cyclase activity [68]. Delling et al. reported that ciliary Ca^2+^ concentrations are significantly higher (≈600 nM) than those found in the cytoplasm (≈100 nM) [20]. Therefore, increased ciliary PC2 could be important for the maintenance of calcium levels in the face of a mechanical stimulus and could maintain cilia length/prevalence downstream of Ca^2+^ dependent adenylyl cyclase activity.

In summary, these findings demonstrate that polycystins play an important role in the chondrocyte response to mechanical stimulation and the regulation of anabolic gene expression. Mechanosensitive trafficking of PC2 to the cilium appears to be an important component of this response and may function to directly regulate cilia-dependent mechanosignalling or as part of a positive feedback loop controlling cilia maintenance and mechanosensitivity. Future studies to further elucidate the mechanistic function of ciliary PC2 have the potential to provide novel cilia targets for drug discovery and promote cartilage health.

## 4. Materials and Methods

### 4.1. Cell and Tissue Culture

Primary bovine articular chondrocytes were isolated from the metacarpal phalangeal joint of freshly slaughtered adult steers (18–24 months). Full depth articular cartilage was dissected from the proximal surface of the joint and subjected to enzymatic digestion overnight as previously described [48]. Primary chondrocytes were cultured in Dulbecco’s modified Eagles Medium (DMEM) supplemented with 10% (*v*/*v*) fetal calf serum (FCS), 1.9 mM l-glutamine, and 96 U/mL penicillin 96 mg/mL streptomycin (Sigma Aldrich, Poole, UK). Cells were seeded at a density of 50,000 cells/cm^2^ and maintained at 37 °C, 5% CO_2_ until confluence. A conditionally immortalized wild-type mouse chondrocyte cell line was cultured as previously described [23]. Murine chondrocytes were maintained in DMEM supplemented with 10% (*v*/*v*) FCS, 88 U/mL penicillin, 90 µg/mL streptomycin, and 2.5 mM-glutamine (Sigma Aldrich). The immortalized cells were maintained at 33 °C, 5% CO_2_ in the presence of 10 nM interferon-γ (IFN-γ, Peprotech, London, UK). For experiments, cells were seeded at 20,000 cells/cm^2^ and cultured under non-permissive conditions at 37 °C without IFN-γ for 4 days. Primary human articular chondrocytes were commercially sourced (Articular engineering, Northbrook, IL, USA). Cells were cultured in chondrocyte growth medium supplemented with 10% human serum (Articular Engineering). Chondrocytes (donors: H-1383 age 78, H-1437 age 70) were maintained at 37 °C, 5% CO_2_ and used at passage 1–3. Cells were seeded at 20,000 cells/cm^2^ and cultured until confluence for experiments.

### 4.2. siRNA Knockdown

Murine chondrocytes were seeded at 20,000 cells/cm^2^ and cultured for 24 h (approximately 50–60% confluence). Cells were transfected with 10 nM siRNA (SilencerSelect^®^ siRNA, Thermo Fisher Scientific, Loughborough, UK) for *pkd1* (s71717) and pkd2 (s233941) and a non-targeting control siRNA (-ve, 4390843) using Lipofectamine RNAi MAX (Thermo Fisher Scientific). Cells were incubated with siRNA made up in optiMEM (Thermo Fisher Scientific) for 48 h.

### 4.3. Application of Cyclic Tensile Strain

Chondrocytes were cultured on collagen type I coated silicone membranes (10 µg/cm^2^) and subjected to 10% uniaxial cyclic tensile strain (CTS) at 0.33 Hz for 0–24 h using both the Flexcell^®^ 5000T system (Protein and RNA isolation) or the Cellscale Mechanoculture FX2 (immunocytochemistry) under serum-free conditions. For unstrained controls, cells were cultured in an identical manner but without the application of strain.

### 4.4. Immunocytochemistry Confocal and Structural Illumination Microscopy

Cells were fixed in 4% paraformaldehyde (PFA) for 10 min followed by permeabilisation in 0.5% Triton X-100. Samples were blocked with 5% donkey serum and then incubated overnight at 4 °C with primary antibodies (Polycystin-2, Santa Cruz Biotechnology, Dallas, TX, USA, sc-25749, 1:200 and a generous gift from Dr. Dominic Norris, MRC Harwell), acetylated α-tubulin (1:2000, T7451, Sigma Aldrich). Samples were washed in phosphate-buffered saline (PBS) and then incubated with Alexa Fluor-conjugated secondary antibodies and 1 µg/mL DAPI (Invitrogen, Carlsbad, CA, USA) for 1 h at room temperature. Samples were mounted with ProLong Diamond mountant (Invitrogen).

Confocal and super resolution microscopy was performed using a Zeiss 710 ELYRA PS.1 microscope (Carl Zeiss, Oberkochen, Germany), 63×/1.4 NA objective. An INCA6600 (GE Healthcare, Chicago, IL, USA) was used for confocal microscopy, 60×/0.75 NA objective. Z-stacks were generated throughout the entire cellular profile using a z-step size of 0.5 μm and reconstructed in a maximum intensity projection for quantification of cilia length, prevalence, and PC2 localization. Cilia were identified by automated imaging analysis, and cilia length and prevalence were determined using Developer Tool box software (GE Healthcare) accompanied by manual validation. For PC2 localization, mean PC2 intensity within the cilium was quantified and normalized to cytoplasmic PC2 expression. A threshold level for PC2 cilia: cytoplasm labeling was manually determined and validated based on no antibody control samples above which cilia were objectively considered to be PC2 positive.

### 4.5. Ca^2+^ Imaging

Murine chondrocytes were seeded onto glass-bottomed dishes and transfected with appropriate siRNA for 48 h. The cells were loaded with 5 µM Fluo-4 AM plus 0.1% Pluronic (Molecular probes) for 15 min at 37 °C and then incubated for a further 15 min at room temperature to allow for dye cleavage. Then, the cells were washed with pre-warmed culture medium. Under these conditions, Fluo-4 brightly labeled every cell examined. Cells were imaged at 63× magnification using confocal microscopy. Samples were imaged every 6 s over a 10 min period (100 cycles). The cells were treated with 100 µM ATP by perfusion after 5 min (cycle 50). The mean fluorescent intensity within individual cells was recorded and plotted against time using Image J software. To objectively identify the proportion of cells exhibiting a Ca^2+^ transient (responder), fluorescence intensity was normalized to the 2 min pre-stimulation period for each cell, and the proportion of responding cells and peak magnitude identified using GraphPad Prism (GraphPad Software, La Jolla, CA, USA). A responding cell was defined as having a peak magnitude greater than 20% above the baseline average, and all responders were manually verified.

### 4.6. RNA Isolation, cDNA Synthesis, and qRT-PCR

Total RNA was isolated and converted to cDNA using the RNeasy mini and Quantitect Reverse Transcription kits (Qiagen, Manchester, UK) according to the manufacturer’s instructions. Quantitative real-time PCR was performed using Syber Green as previously described [39]. Primer sequences were GAPDH: F-GACAAAATGGTGAAGGTCGG R-TCCACGACATACTCAGCACC, Acan: F-CACGCTACACCCTGGACTTTG R-CCATCTCCTCAGCGAAGCAGT Col2a: F-GGCAACAGCAGGTTCACATA R-ATGGGTGCGATGTCAATAAT.

### 4.7. Protein Isolation and Western Analyses

For protein isolation cells were cultured in 6-well plates; at the end of the experiment, cells were briefly washed in ice-cold PBS followed by incubation with 300 µL ice-cold RIPA buffer (Sigma Aldrich) containing a cocktail of protease inhibitors (Roche). The cells were scraped from the culture surface and incubated on ice for 15 min; then, they were homogenized through a 21 G needle. Samples were centrifuged at 8000× *g* for 15 min at 4 °C and the supernatant was transferred to a fresh tube and frozen for later quantification and use. Proteins were resolved using Mini-PROTEAN TGX^TM^ Precast gels (Biorad, Watford, UK) and transferred to nitrocellulose membranes using the TransBlot Turbo system (Biorad). Membranes were incubated in primary antibodies overnight (PC2, Santa Cruz Biotechnology, Sc-25749), beta-actin (Abcam, Ab8226) and immunoreactive bands were labeled using LI-COR near-infrared secondary antibodies and quantified using Li-Cor Image Studio™ Lite.

### 4.8. Data Presentation and Statistical Analyses

Graphs were prepared using GraphPad Prism (GraphPad Software, La Jolla, CA, USA). Statistical analyses were performed in GraphPad Prism. Statistical significance is represented as * *p* < 0.05, ** *p* < 0.01, and *** *p* < 0.001. Data were assessed for normality prior to analyses, for data found to be not normal, Box-Cox transformation was performed prior to analyses. The statistical analyses and experimental n numbers used are described in the respective figure legends (typically: one-way ANOVA, two-way ANOVA, and Chi-squared test). For experiments using murine cell lines, experiments were minimally performed in triplicate, whereas for primary chondrocytes, experiments were conducted with a minimum of 3 donors unless otherwise stated. Data are presented as mean ± standard deviation (SD) unless otherwise stated.

## Data Availability

Requests for data, resources and reagents should be directed to the co-corresponding author Clare L. Thompson (clare.l.thompson@qmul.ac.uk).

## Supplementary Materials

The following are available online at https://www.mdpi.com/article/10.3390/ijms22094313/s1, Figure S1: Donor variability in Polycystin-2 localization, Figure S2: Polycystin-2 localizes to the cilium in WT and ORPK chondrocytes.
