# Supplementary material for: Polycystin-2 Is Required for Chondrocyte Mechanotransduction and Traffics to the Primary Cilium in Response to Mechanical Stimulation
Source: Int J Mol Sci. 2021 Apr 21;22(9):4313. doi: 10.3390/ijms22094313 (PMC8122406; doi:10.3390/ijms22094313)
Supplement: Supplementary file 1 [file ijms-22-04313-s001.zip › ijms-1165639-supplementary.pdf]

**Polycystin-2 is required for chondrocyte mechanotransduction and traffics to the primary cilium in response to mechanical stimulation**
Clare L Thompson[1*], Megan Mc Fie[1] J Paul Chapple[2], Hannah M Mitchison[3], Philip Beales[3], Martin M Knight[1]

[1]Centre for Predictive *In Vitro* Models, School of Engineering and Materials Science, Queen Mary University of London, London, E1 4NS, UK. [2] Centre for Endocrinology, William Harvey Research Institute, School of Medicine and Dentistry, Queen Mary University of London, London EC1M 6BQ, UK. [3] Genetics and Genomic Medicine, UCL Great Ormond Street Institute of Child Health, London WC1N 1EH, UK

## Supplementary figures

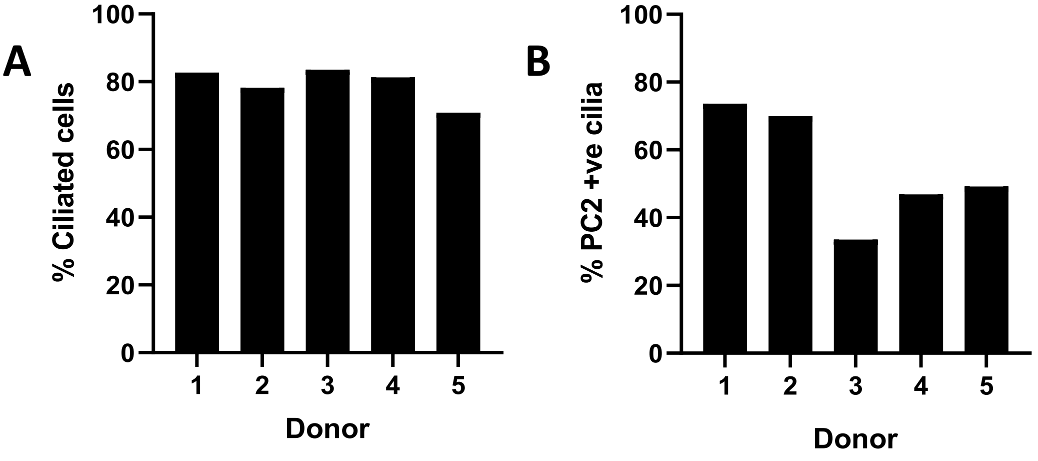


## Figure S1: Donor variability in Polycystin-2 localisation

Freshly isolated bovine articular chondrocytes were cultured on glass coverslips until confluence. Chondrocytes were immuno-labelled for polycystin-2 (PC2) , primary cilia were labelled for acetylated tubulin and nuclei were counterstained with DAPI. Both the proportion of (A) ciliated cells and (B) PC2 positive cilia was quantified.

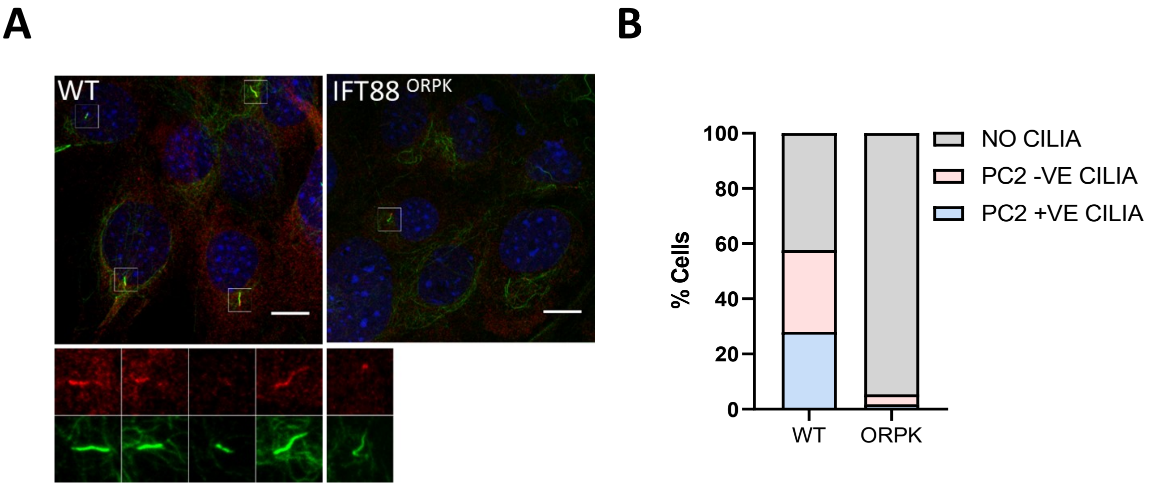


## Figure S2: Polycystin-2 localises to the cilium in WT and ORPK chondrocytes

(A) Immortalised murine chondrocytes (WT and IFT88$^{ORPK}$ mutant) were labelled for PC2 (red), acetylated tubulin (green) and nuclei counterstained with DAPI (Blue). Scale bar 5µm. The proportion of ciliated cells and PC2 positive cilia was determined (n>100 cilia).
